# Supplementary figures and images for: RNA structure prediction using positive and negative evolutionary information
Source: PLoS Comput Biol. 2020 Oct 30;16(10):e1008387. doi: 10.1371/journal.pcbi.1008387 (PMC7657543; doi:10.1371/journal.pcbi.1008387)

# transfer messenger RNA

a

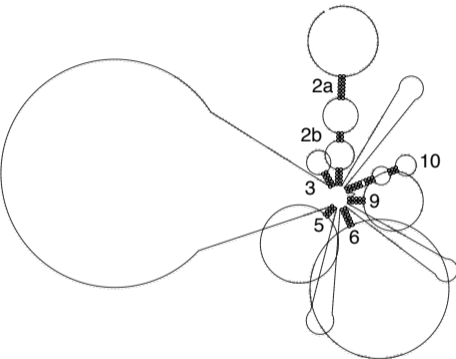

RNAalifold

b

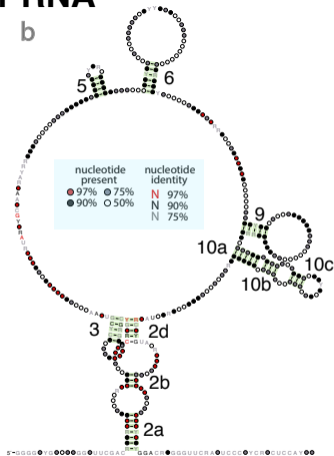

RNAalifold & R-scape

Supplement: S1 Fig — (a) The RNAalifold predicted consensus structure output for the tmRNA Rfam seed alignment (RF00023) obtained using default parameters. The RNAalifold structure consists of 46 basepairs, and it annotates (at least partially) 6 of the 12 helices in the structure [44]: 2 (a,b,d), 3, 5, 6, 9, and 10 (a,b,c), see Fig 3g. (b) The covariation analysis of the RNAalifold structure indicates that 45 of the 46 RNAalifold basepairs have covariation support (shown in green). It also identifies 76 other basepairs with covariation support not in the proposed RNAalifold structure. The display of all 121 positive pairs can be seen in Fig 3f. (Columns with more than 75% gaps have been removed from the display.). (PDF) [file pcbi.1008387.s001.pdf]

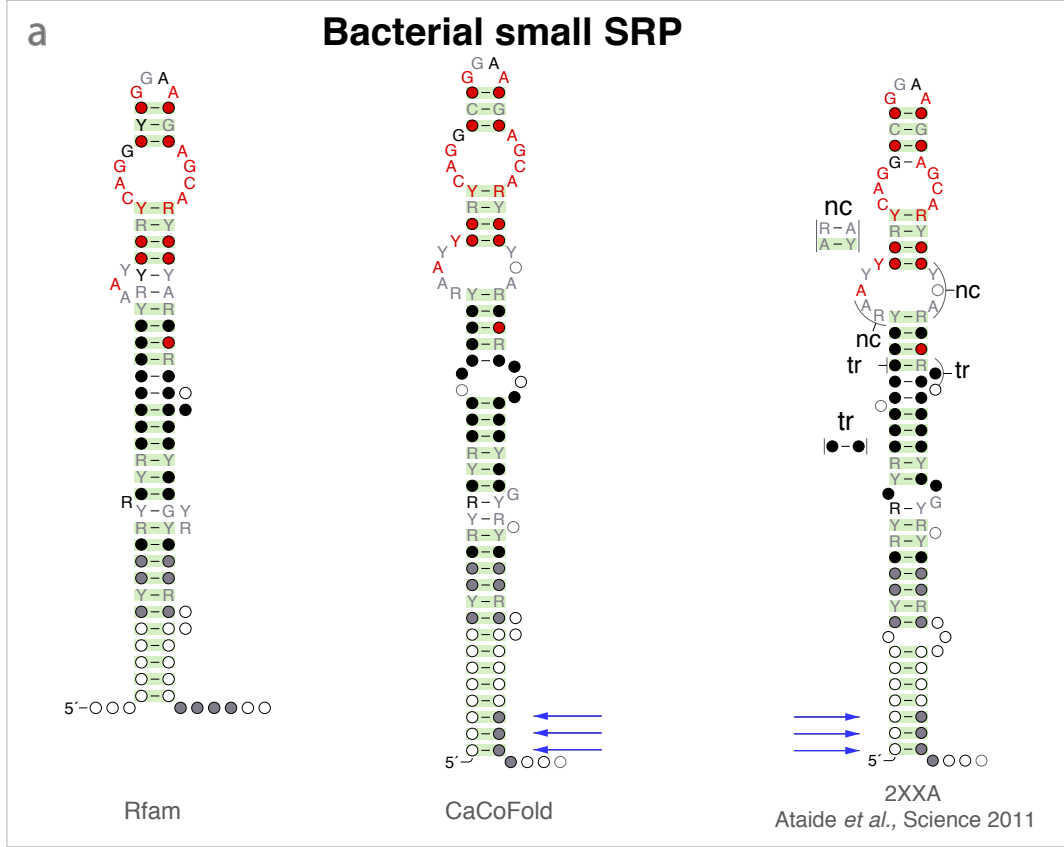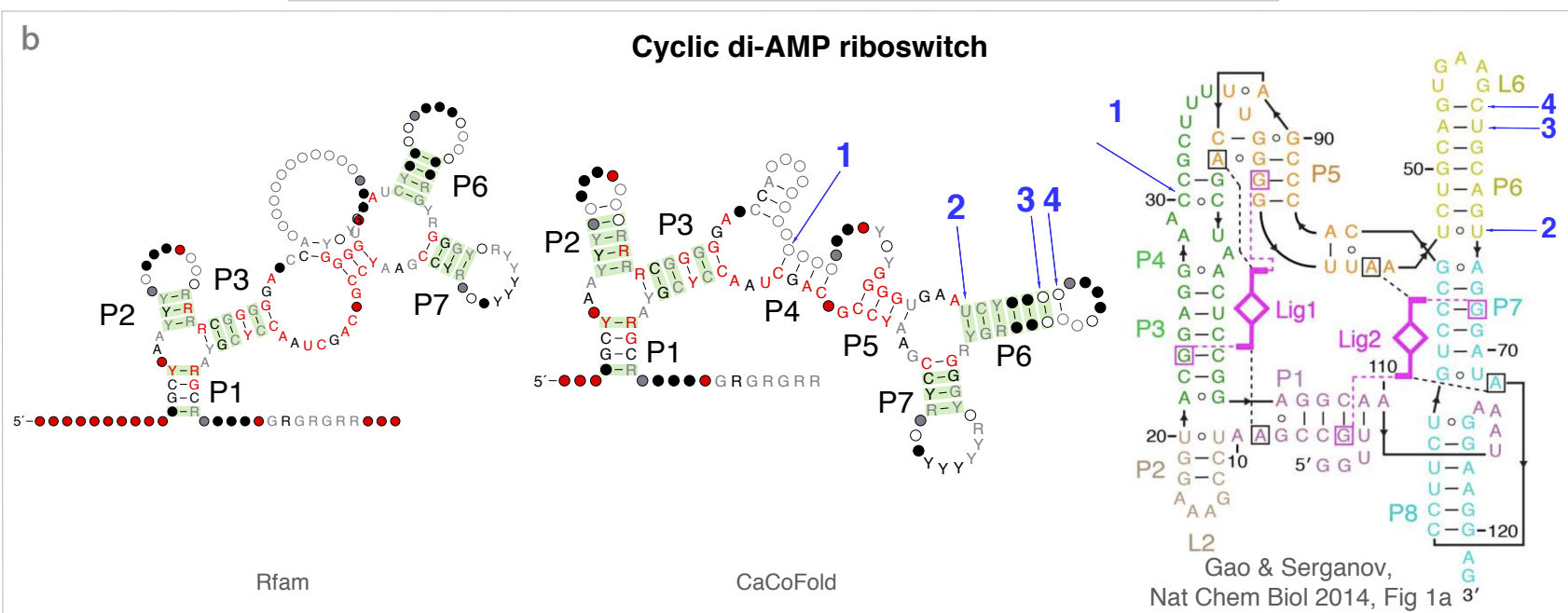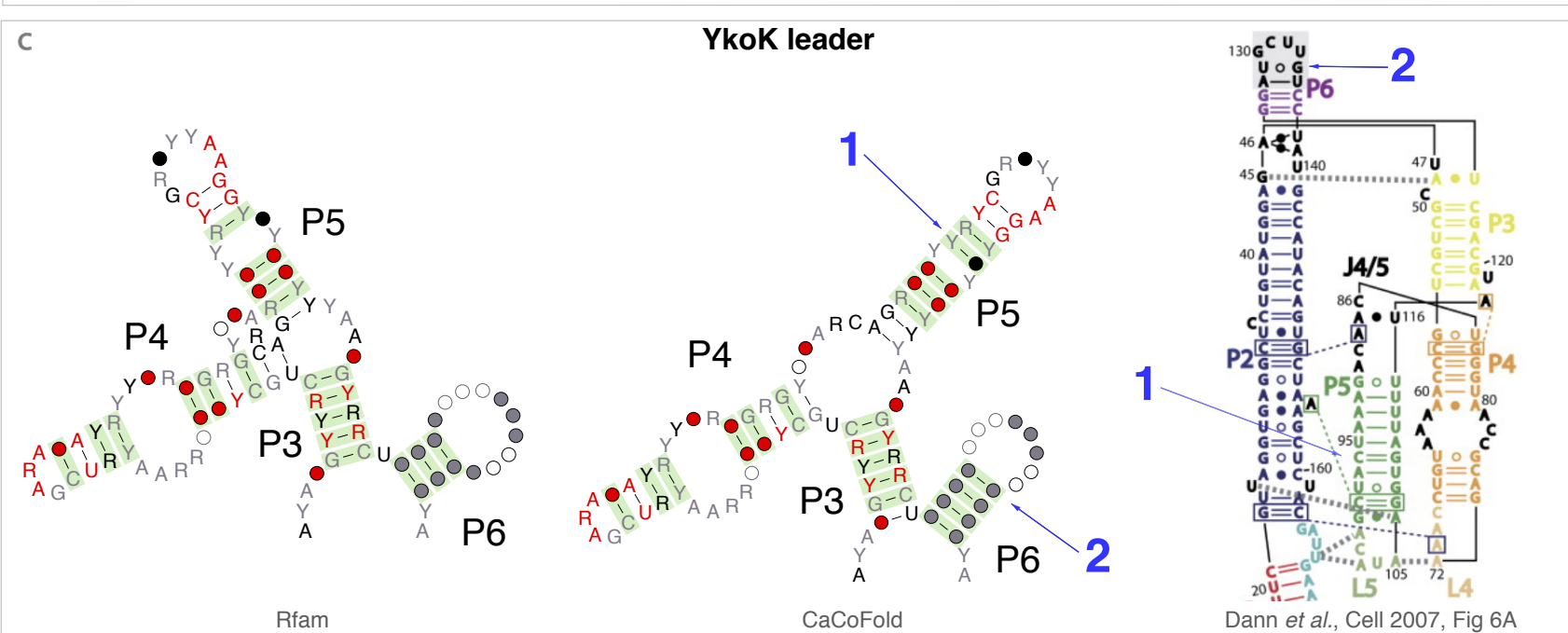

Supplement: S2 Fig — Structural elements with covariation support introduced by CaCoFold relative to the Rfam annotation and corroborated by 3D structures are annotated in blue. The three RNAs are examples of CaCoFold structures with more covariation support in the form of more positive basepairs to helices already present in the consensus Rfam structures. (a) SRP RNA. The SRP complex 2XXA PDB X-ray diffraction structure has 3.94 Å resolution [54]. The PDB-derived consensus structure was obtained as described in Methods. (b) For the cyclic di-AMP riboswitch, the region around helix P4 is highly variable in the Rfam alignment, and none of the proposed structures has covariation support. The displayed CaCoFold structure showing helix P4 was obtained using a consensus reference sequence (instead of the default profile sequence). The rest of the structure has covariation support and remains invariant. (c) For the YkoK leader, there are two additional basepairs labeled “1” and “2” in helices P5 and P6 respectively confirmed by the crystal structure [56]. (PDF) [file pcbi.1008387.s002.pdf]

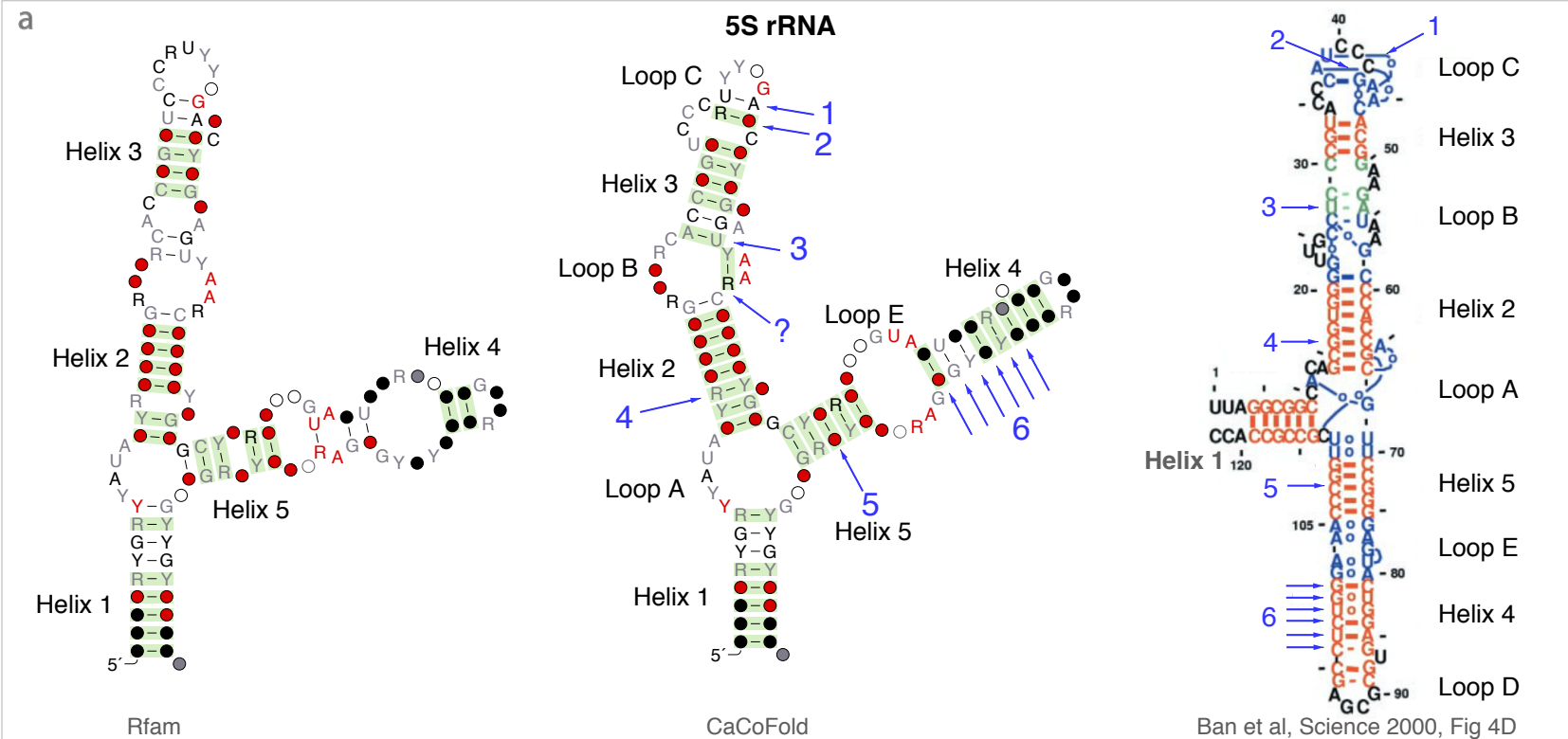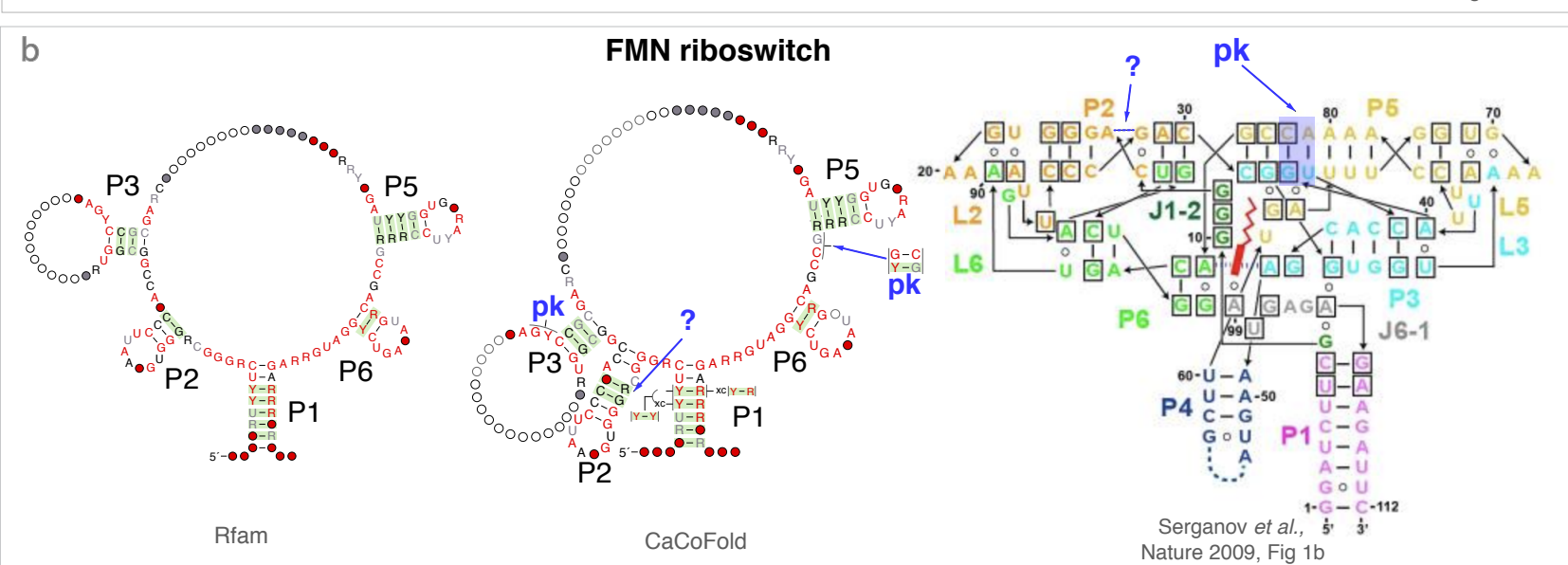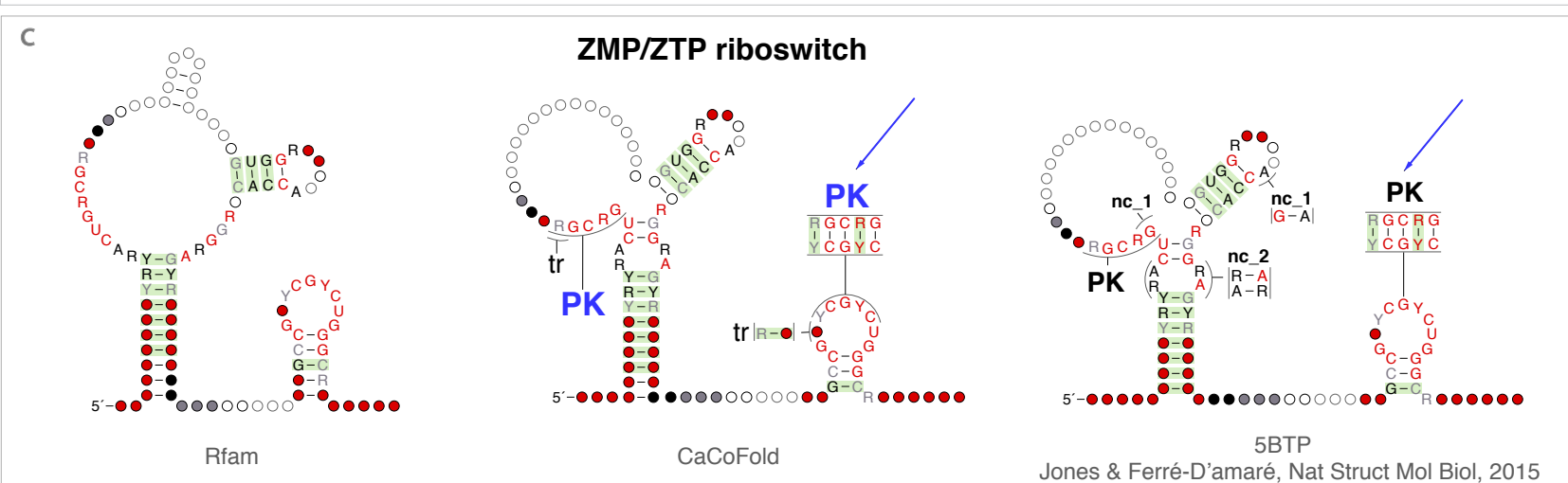

Supplement: S3 Fig — Structural elements with covariation support introduced by CaCoFold relative to the Rfam annotation and corroborated by 3D structures are annotated in blue. (a) The 5S rRNA CaCoFold structure remodels Helix 4 (six basepairs) and Loop C (two basepairs) in agreement with the crystal structure [57]. A Y-R covarying basepair in Loop B is not described in the 3D structure. (b) The FMN riboswitch CaCoFold structure identifies a confirmed 2-basepair pseudoknotted helix, and one covarying pair in helix P2 that is different than in the 3D structure [58]. (c) The covarying pseudoknot identified by CaCoFold in the ZPM-ZTP riboswitch is confirmed by the Fusobacterium ulcerans X-ray diffraction structure (2.82 Å) [59]. (PDF) [file pcbi.1008387.s003.pdf]

a

Fluoride riboswitch

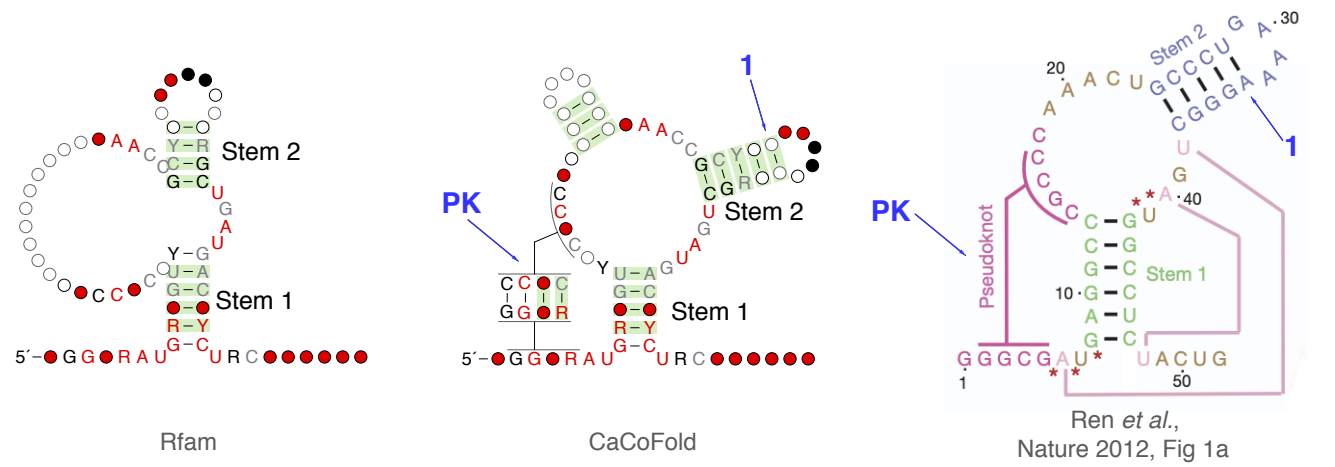

b

Glutamine riboswitch

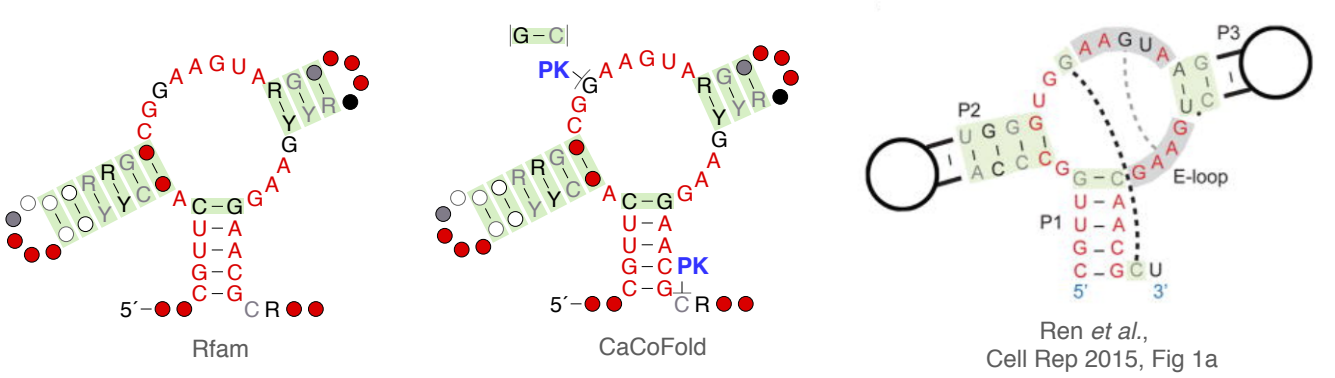

c

Archaea SRP

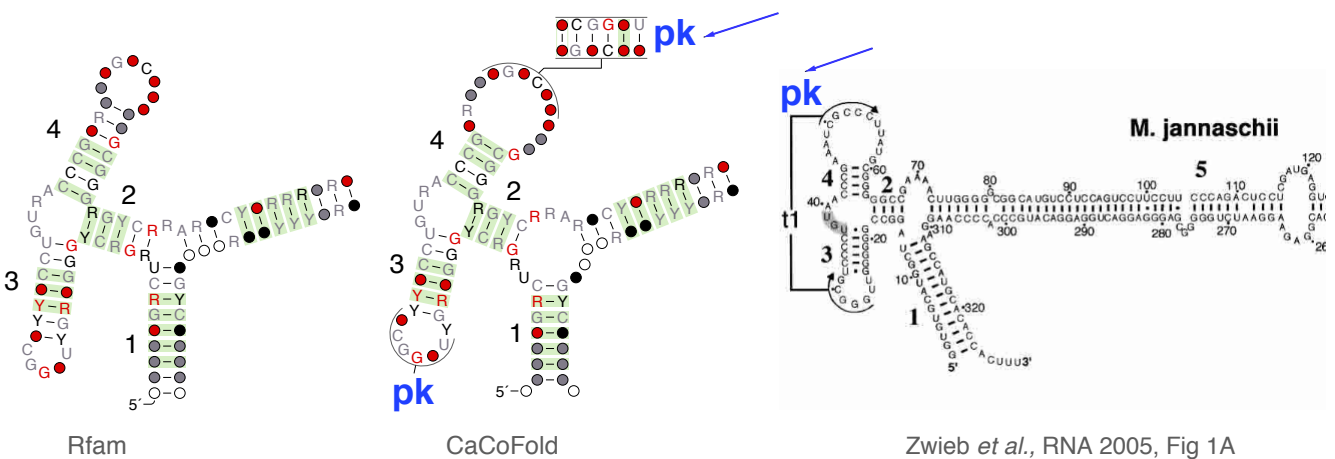

Supplement: S4 Fig — Structural elements with covariation support introduced by CaCoFold relative to the Rfam annotation and corroborated by 3D structures are annotated in blue. All three cases (a) Fluoride riboswitch (b) Glutamine riboswitch (c) Archeal SRP are examples of CaCoFold structures with more covariation support in the form of a new helix forming a pseudoknot all confirmed by the 3D structures. (PDF) [file pcbi.1008387.s004.pdf]

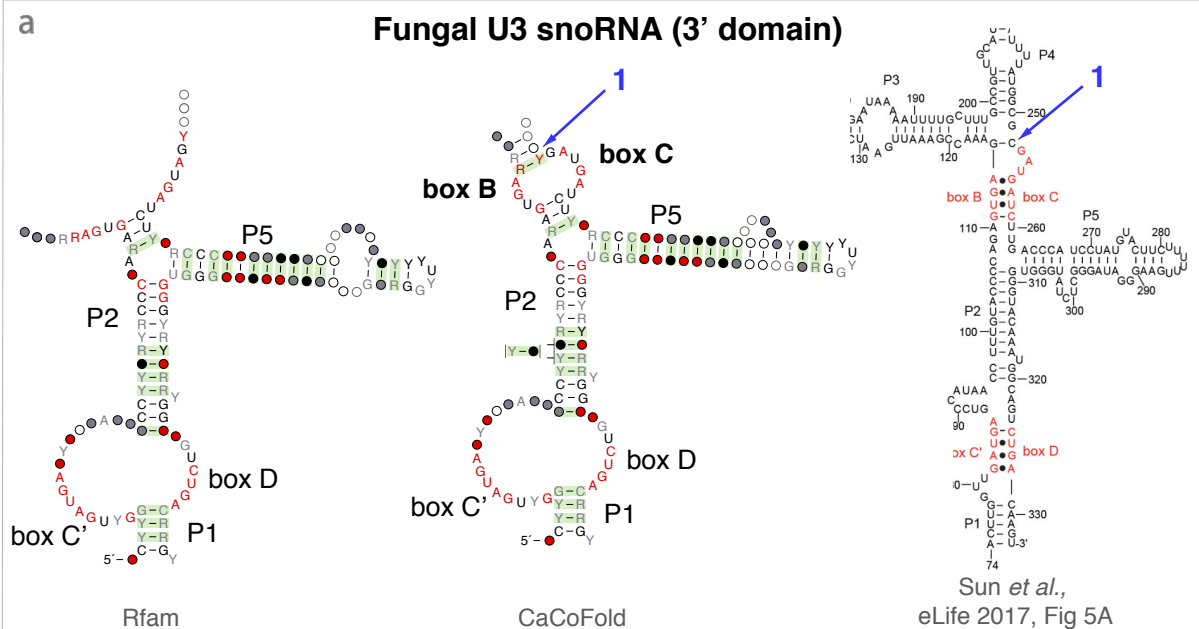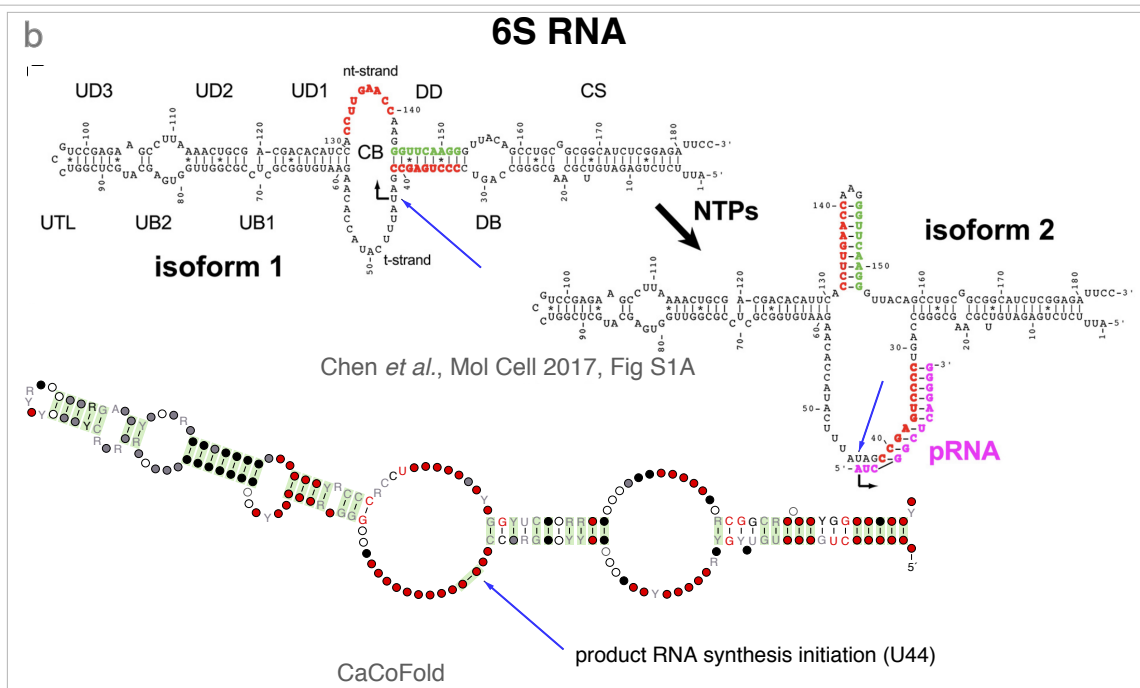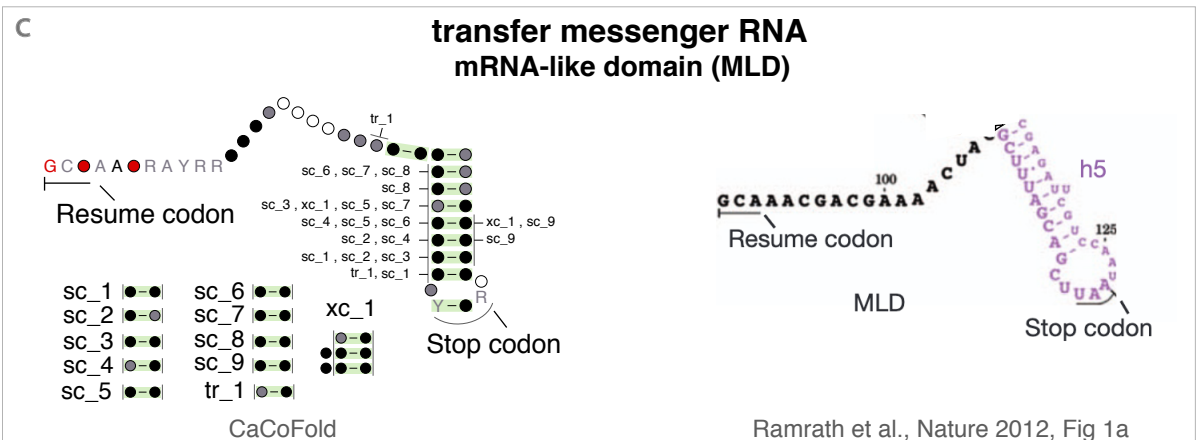

Supplement: S6 Fig — Structural elements with covariation support introduced by CaCoFold relative to the Rfam annotation and corroborated by 3D structures are annotated in blue. (a) The U3 snoRNA CaCoFold structure adds a covarying pair closing the boxB/boxC of the snoRNA [65]. (b) 6S RNA covarying pair at the RNA synthesis initiation site not associated to RNA structure [4]. (c) Side-covariation in the mRNA-like domain of tmRNA not due to RNA structure. (PDF) [file pcbi.1008387.s006.pdf]

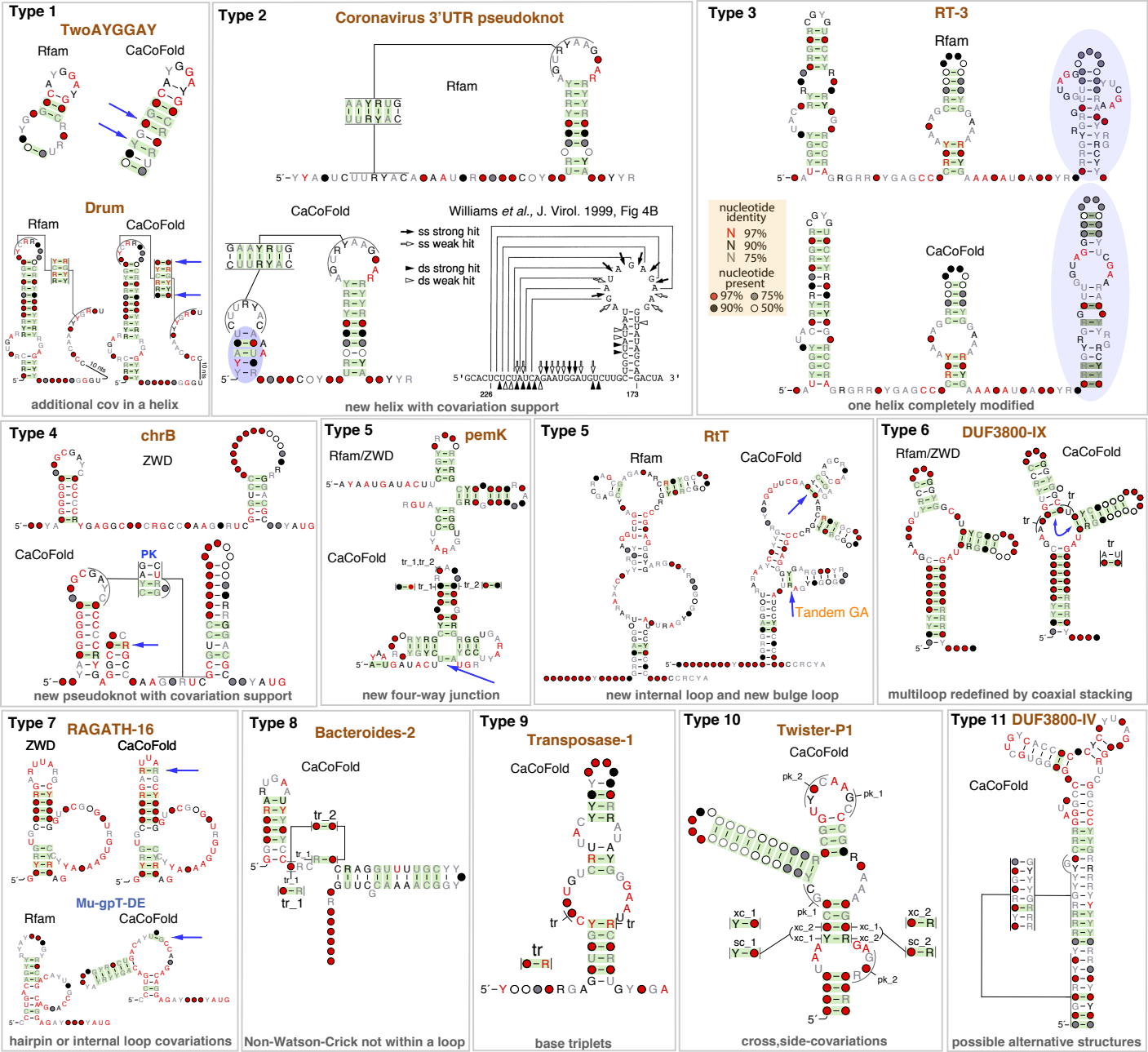

Supplement: S7 Fig — We provide examples of differences corresponding to Types 1 to 11. A description of all different types is given in Table 1. (PDF) [file pcbi.1008387.s007.pdf]
