# Supplementary material for: RNA structure prediction using positive and negative evolutionary information
Source: PLoS Comput Biol. 2020 Oct 30;16(10):e1008387. doi: 10.1371/journal.pcbi.1008387 (PMC7657543; doi:10.1371/journal.pcbi.1008387)
Supplement: S5 Fig — Structural elements with covariation support introduced by CaCoFold relative to the Rfam annotation and corroborated by 3D structures are annotated in blue. (a) An additional covarying pair introduces a new internal loop in the B-type RNase P RNA confirmed by Ref. 63, Fig 4a. (b) An additional covarying pair introduces a new three-way junction in the Group-II intron D1D4-3 fragment [64]. (c) In the U5 snRNA, an additional Y-Y covarying pair that modifies a hairpin loop is confirmed by the S. pombe spliceosomal RNA cryo-EM structure 3JB9 (3.60 Å) [65]. (PDF) [file pcbi.1008387.s005.pdf]

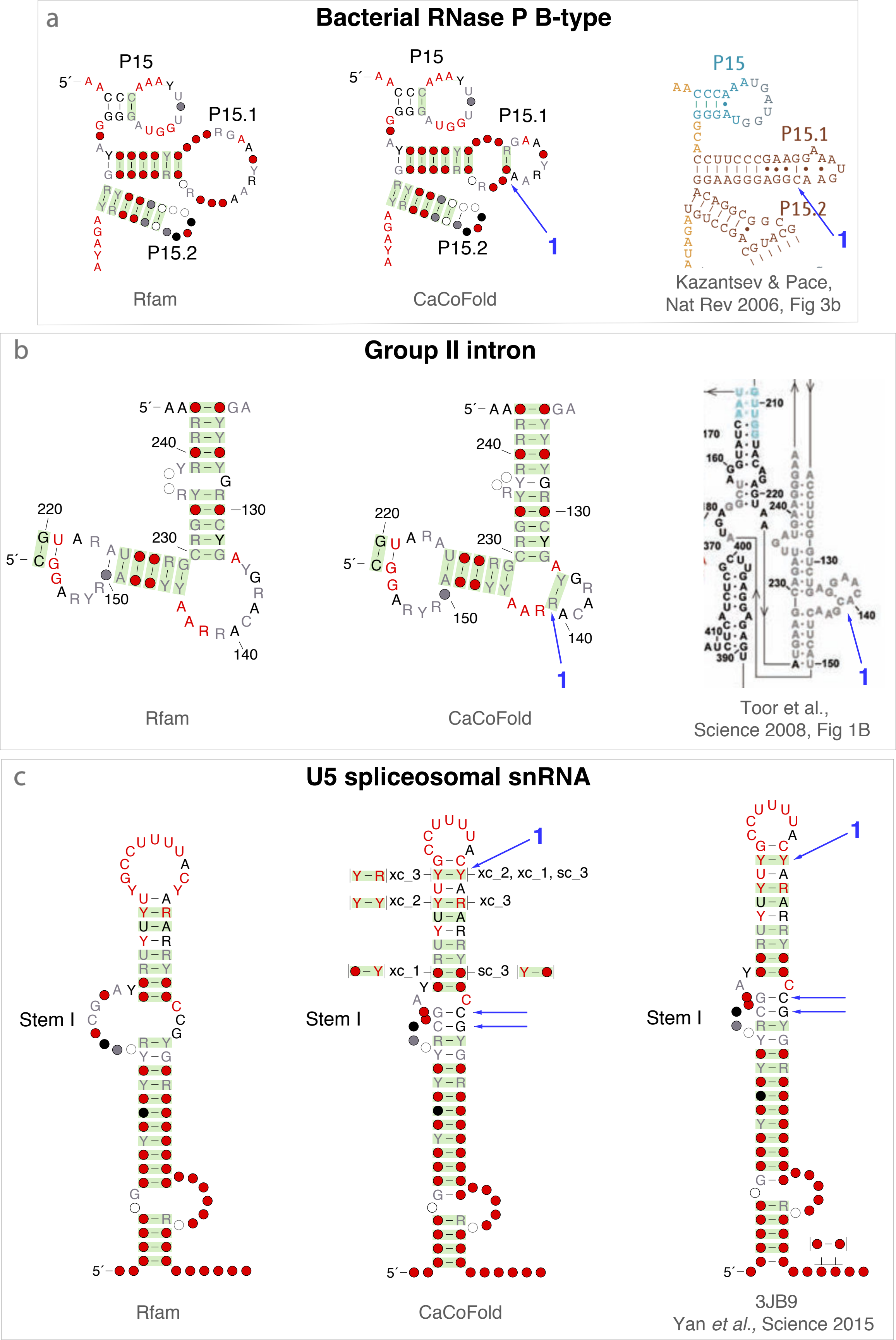
a
Bacterial RNase P B-type
P15
P15.1
P15.2
5´–
Rfam
CaCoFold
Kazantsev & Pace,
Nat Rev 2006, Fig 3b
1
b
Group II intron
220
240
230
130
150
140
Rfam
CaCoFold
Toor et al.,
Science 2008, Fig 1B
1
c
U5 spliceosomal snRNA
Stem I
xc_3
xc_2, xc_1, sc_3
xc_2
xc_3
xc_1
sc_3
Rfam
CaCoFold
3JB9
Yan et al., Science 2015
1
