# Supplementary material for: RNA structure prediction using positive and negative evolutionary information
Source: PLoS Comput Biol. 2020 Oct 30;16(10):e1008387. doi: 10.1371/journal.pcbi.1008387 (PMC7657543; doi:10.1371/journal.pcbi.1008387)
Supplement: S1 Table — (PDF) [file pcbi.1008387.s009.pdf]

Table S1. **277 Rfam and 105 ZWD structural alignments for which CaCoFold proposes a structure including larger number of covarying basepairs.** List of alignments for which the CaCoFold structure incorporates more covarying pairs. The alignments have been classified as belonging to one of 15 types described in the main text depending on what kind of modifications the CaCoFold structure introduces. Each alignment has been classified into one type. Here we note alignments that could be placed into more than one category. Because many of the RNAs in ZWD have been incorporated into Rfam, there is an important overlap between the two databases. The 382 structural alignments (277 Rfam + 105 ZWD) with improved CaCoFold structure represent 314 different RNAs, as 68 of the 105 ZWD RNAs have been incorporated into Rfam v14.1. For the 68 RNAs with representation in both databases, the corresponding alignments and structures are sometimes different. These 68 RNAs with different Rfam and ZWD alignments have been grouped together when the structural changes introduced by CaCoFold correspond to the same type. They are only 5/68 RNAs (PhotoRC-II/RF01717, manA/RF01745, radC/RF01754, pemK/RF02913, Mu-gpT-DE/RF03012) for which the ZWD and Rfam v14.1 alignments resulted in different CaCoFold structural modifications. All alignments with the structural annotation are provided as part of the supplemental materials.

Type 1: Helix expanded by additional covariations

		Rfam alignment				ZWD alignment				
RNA	Rfam ID	extra covs in CaCoFold structure	CaCoFold structure cov_bps/bps	Rfam structure cov_bps/bps	covs obs/exp	extra covs in CaCoFold structure	CaCoFold structure cov_bps/bps	ZWD structure cov_bps/bps	covs obs/exp	other types
PhotoRC-II	RF01717	7	20/24	13/13	20/20.8	1	17/21	16/19	17/15.4	
6A	RF02925	1	11/14	10/10	11/8.7					
TwoAYGGAY	RF01731	3	45/56	42/45	45/46.6					
drum	RF02958	3	29/38	26/28	29/33.7	2	26/34	24/28	26/30.7	
group-II-D1D4-7	RF02012	2	40/42	38/41	40/41.4	2	43/45	41/44	43/44.2	
T-box	RF00230	1	41/51	40/47	41/28.4					
Bacteria small SRP	RF00169	3	31/31	28/33	31/30.9					
DUF2800	RF02960	1	32/53	31/40	32/28.5	1	31/53	30/46	31/30.3	
malK-I	RF03069	1	40/52	39/54	40/40.7					
Flavobacterium-1	RF02976	1	24/36	23/37	24/16.3	1	24/36	23/37	24/17.1	
ykoK	RF00380	2	31/44	29/47	31/36.4					
DUF3800-III	RF02962	1	19/34	18/31	19/9.6	1	19/34	18/31	19/10.1	
ydaO-yuaA	RF00379	3	18/37	15/26	18/22.3					
terC	RF03067	1	9/16	8/15	9/7.8	1	9/16	8/15	9/7.9	
RT-4	RF03025	1	16/30	15/29	16/5.9	1	16/30	15/30	16/6.6	
Betaproteobacteria toxic sRNA	RF02278	1	8/17	7/16	8/5.0					
engA	RF02972	1	8/25	7/27	8/5.2	1	8/26	7/27	8/5.9	
DUF3800-XI	RF02975	1	3/14	2/10	3/5.7					
mir-929	RF00947	1	3/31	2/26	3/1.6					
group-II-D1D4-8						1	33/37	32/38	33/35.7	
group-I-patch3						2	30/35	28/36	30/29.3	9
HEARO	RF02033					1	60/85	59/85	60/52.7	
c-di-GMP-II-GAG	RF01786					1	19/28	18/29	19/21.4	

Type 2: One new helix with covariation support

		Rfam alignment				ZWD alignment				
RNA	Rfam ID	extra covs in CaCoFold structure	CaCoFold structure cov_bps/bps	Rfam structure cov_bps/bps	covs obs/exp	extra covs in CaCoFold structure	CaCoFold structure cov_bps/bps	ZWD structure cov_bps/bps	covs obs/exp	other types
group-II-D1D4-4	RF02003	2	26/37	24/27	26/29.9	1	25/29	24/27	25/10.8	
yybP-ykoY	RF00080	1	18/59	17/25	18/21.2					
sok	RF01794	3	17/51	14/27	17/31.1					8
Mahella-1	RF03007	1	9/31	8/22	9/2.1	1	9/31	8/23	9/2.2	
Streptomyces-metH	RF03076	1	4/36	3/13	4/7.8	1	4/36	3/13	4/7.9	
snoZ152	RF00350	1	2/18	1/5	2/0.6					
MIR1446	RF00954	1	4/36	3/36	4/0.3					
Afu 254	RF01504	1	2/11	1/12	2/0.4					
HgcC	RF00062	3	4/33	1/27	4/0.7					9
beta tmRNA	RF01850	1	3/107	2/55	3/2.9					
ldcC						1	9/15	8/11	9/10.0	

Type 3: One helix completely modified

		Rfam alignment				ZWD alignment				
RNA	Rfam ID	extra covs in CaCoFold structure	CaCoFold structure cov_bps/bps	Rfam structure cov_bps/bps	covs obs/exp	extra covs in CaCoFold structure	CaCoFold structure cov_bps/bps	ZWD structure cov_bps/bps	covs obs/exp	other types
5S rRNA	RF00001	9	31/33	22/34	31/32.4					
RT-3	RF03068	8	28/41	20/33	28/21.2					
manA	RF01745	19	48/65	29/59	48/48.8	1	55/68	54/63	55/36.2	
IS605-orfB-I	RF03065	14	40/66	26/63	40/39.0					
mir-17	RF00051	1	3/31	2/25	3/1.2					
sraA	RF02029	1	2/19	1/20	2/1.3					
P18	RF01671	1	2/38	1/41	2/0.1					

Type 4: New pseudoknot with covariation support

		Rfam alignment				ZWD alignment				
RNA	Rfam ID	extra covs in CaCoFold structure	CaCoFold structure cov_bps/bps	Rfam structure cov_bps/bps	covs obs/exp	extra covs in CaCoFold structure	CaCoFold structure cov_bps/bps	ZWD structure cov_bps/bps	covs obs/exp	other types
Coronavirus 3'UTR pk	RF00165	2	16/22	14/15	16/8.7					
gut-2	RF02990	1	12/18	11/12	12/8.2	1	12/18	11/12	12/8.4	
U2	RF00004	6	45/54	39/45	45/43.5					9
Fluoride	RF01734	6	14/17	8/10	14/13.6					5
SAM	RF00162	8	27/35	19/27	27/27.5					6
radC	RF01754	2	24/42	22/35	24/29.8	1	26/56	25/40	26/26.2	
GOLLD	RF02032	13	79/139	66/106	79/66.2					
FMN	RF00050	4	17/40	13/23	17/23.1					10,1
Archaea SRP	RF01857	1	69/111	68/132	69/63.5					
ZMP-ZTP	RF01750	4	15/25	11/22	15/16.3					
chrB-a	RF03078	1	5/19	4/12	5/6.0					2
STAXI	RF01728	5	15/51	10/33	15/16.6					
snoZ30	RF00288	1	3/34	2/7	3/1.9					
ToxI	RF02519	1	2/8	1/4	2/0.0					
snR49	RF01239	5	7/50	2/47	7/0.3					9
sRNA162	RF02792	1	2/51	1/50	2/0.0					

TB10Cs4H2	RF01862	2	2/21	0/16	2/0.0					

Type 5: New junction or new internal loop or new coaxials

		Rfam alignment				ZWD alignment				
RNA	Rfam ID	extra covs in CaCoFold structure	CaCoFold structure cov_bps/bps	Rfam structure cov_bps/bps	covs obs/exp	extra covs in CaCoFold structure	CaCoFold structure cov_bps/bps	ZWD structure cov_bps/bps	covs obs/exp	other types
pemK	RF02913	10	25/25	15/15	25/24.3	4	18/18	14/15	18/17.5	
RAGATH-18	RF03064	10	27/28	17/18	27/27.2	7	24/25	17/18	24/24.3	7
cow-rumen-2	RF02947	3	19/27	16/17	19/6.7	3	19/27	16/17	19/6.8	4
Cobalamin	RF00174	14	49/56	35/42	49/47.4					1,9,7
RNaseP bact a	RF00010	7	101/115	94/114	101/104.8					9,7
U4	RF00015	2	27/40	25/31	27/32.6					
group-II-D1D4-5	RF02004	3	46/58	43/54	46/51.3	3	43/59	40/54	43/37.8	
group-II-D1D4-3	RF02001	3	39/41	36/48	39/39.7	2	38/41	36/48	38/37.8	
SpF59 sRNA	RF02440	2	11/16	9/12	11/10.8					2
RNaseP bact b	RF00011	2	76/104	74/105	76/71.8					
OLE	RF01071	2	51/187	49/159	51/39.6					2
RtT	RF00391	2	8/33	6/24	8/11.0					1
MIR169 2	RF00645	4	9/37	5/22	9/20.9					9
freshwater-1	RF02979	1	4/14	3/15	4/0.4					
rsmX	RF02144	1	3/32	2/36	3/2.8					
vWFA-era						1	12/16	11/16	12/1.8	
c-di-GMP-I-UAC	RF01051					1	14/23	13/22	14/8.5	

Type 6: Internal loop or multiloop reshaped by coaxial stacking

		Rfam alignment				ZWD alignment				
RNA	Rfam ID	extra covs in CaCoFold structure	CaCoFold structure cov_bps/bps	Rfam structure cov_bps/bps	covs obs/exp	extra covs in CaCoFold structure	CaCoFold structure cov_bps/bps	ZWD structure cov_bps/bps	covs obs/exp	other types
DUF3800-IX	RF02968	4	22/27	18/21	22/21.2	4	23/28	19/22	23/23.0	
group-II-D1D4-6	RF02005	5	51/61	46/58	51/54.2	3	49/68	46/58	49/38.3	
RT-2	RF03077	3	38/56	35/45	38/43.2	3	41/58	38/48	41/46.3	
manA	RF01745	19	48/65	29/59	48/48.8	1	55/68	54/63	55/36.2	
Fungi U3	RF01846	3	39/101	36/79	39/63.9					1,10
potC	RF01751	1	4/17	3/17	4/6.8	1	4/18	3/17	4/3.3	
TwoGGAY						3	46/55	43/46	46/42.2	9
RAGATH-2-HDV						3	26/28	23/26	26/27.0	
hatchet						5	34/61	29/37	34/40.0	
group-I-patch5						1	19/23	18/23	19/19.3	
group-II-D1D4-9						2	76/140	74/122	76/73.0	7
IS605-orfB						2	42/65	40/69	42/40.2	1

Type 7: Hairpin or internal loop covariations (often non Watson-Crick)

		Rfam alignment				ZWD alignment				
RNA	Rfam ID	extra covs in CaCoFold structure	CaCoFold structure cov_bps/bps	Rfam structure cov_bps/bps	covs obs/exp	extra covs in CaCoFold structure	CaCoFold structure cov_bps/bps	ZWD structure cov_bps/bps	covs obs/exp	other types
int-alpA	RF02996	7	30/32	23/23	30/26.3	11	34/35	23/23	34/30.7	
RAGATH-16	RF03047	1	14/18	13/16	14/2.3	1	13/17	12/16	13/2.3	
DUF3800-VIII	RF03075	1	19/25	18/25	19/18.0	1	20/29	19/25	20/22.5	
alpha tmRNA	RF01849	1	33/75	32/56	33/49.8					
DUF3577	RF02954	1	14/32	13/23	14/10.4					
Mu-gpT-DE	RF03012	2	7/12	5/9	7/6.5	2	12/13	10/11	12/10.7	8
RT-12	RF03016	1	18/46	17/40	18/10.2	1	18/46	17/43	18/10.1	
Actino-pnp	RF01688	2	11/27	9/22	11/13.3	1	16/23	15/26	16/10.4	
IMES-2	RF02035	1	16/32	15/39	16/20.2	1	18/46	17/44	18/16.8	
poplar-1	RF03041	1	13/37	12/32	13/3.9	1	13/37	12/32	13/3.9	
traJ-II	RF01760	1	13/34	12/33	13/13.4	1	12/33	11/33	12/6.9	
DUF3085	RF02959	1	6/20	5/18	6/1.2					
RAGATH-13	RF02688	1	2/25	1/23	2/0.7	1	1/25	0/23	1/0.7	
McaS	RF00115	1	2/26	1/24	2/0.1					
CopA	RF00042	6	7/34	1/25	7/6.3					
mir-3	RF00716	1	2/23	1/26	2/1.0					
mir-344	RF00825	1	2/26	1/32	2/4.8					
group-II-D5D6-1						1	19/34	18/29	19/17.7	
RAGATH-34	RF03103					1	8/25	7/27	8/2.2	

Type 8: Non Watson-Crick (not within a loop) covariations

		Rfam alignment				ZWD alignment				
RNA	Rfam ID	extra covs in CaCoFold structure	CaCoFold structure cov_bps/bps	Rfam structure cov_bps/bps	covs obs/exp	extra covs in CaCoFold structure	CaCoFold structure cov_bps/bps	ZWD structure cov_bps/bps	covs obs/exp	other types
tRNA	RF00005	16	37/37	21/21	37/37.0					
Rhodo-rpoB	RF03074	1	22/26	21/23	22/21.3	1	22/26	21/23	22/21.7	
6S	RF00013	1	40/49	39/49	40/41.2					
glnA	RF01739	1	11/15	10/14	11/11.0					
THF	RF01831	2	26/33	24/36	26/25.2					
Mu-gpT-DE	RF03012	2	7/12	5/9	7/6.5	2	12/13	10/11	12/10.7	8
RAGATH-27	RF03100	1	9/23	8/15	9/1.9	1	9/22	8/15	9/1.8	
Corona FSE	RF00507	1	5/24	4/19	5/1.0					
psaA	RF01752	1	10/60	9/62	10/12.9	1	10/60	9/63	10/6.6	
Atu C6	RF02500	1	4/35	3/25	4/6.8					
snR8	RF01248	1	5/40	4/34	5/1.7					
Atu C8	RF02502	1	4/32	3/32	4/4.2					
Y RNA	RF00019	1	3/23	2/23	3/7.1					
SAM-II long loops	RF01726	1	2/5	1/12	2/1.5					
Rhino CRE	RF00220	1	2/24	1/21	2/0.5					
snoR80	RF01224	1	2/28	1/24	2/0.8					
MIR405	RF00768	1	2/42	1/30	2/0.7					
IMPDH	RF02994	1	2/31	1/31	2/3.2	1	2/31	1/31	2/3.5	
freshwater-2	RF02980					2	14/19	12/17	14/12.7	
Bacteroides-2						4	15/23	11/19	15/6.0	
HTH-XRE	RF02923					1	6/19	5/13	6/1.9	
RAGATH-24	RF03049					1	10/42	9/40	10/3.6	
Ocean-VI	RF01714					3	5/33	2/23	5/6.2	
Chloroflexus-1	RF02936					2	3/59	1/43	3/0.0	

Type 9: Triplets

		Rfam alignment				ZWD alignment				
RNA	Rfam ID	extra covs in CaCoFold	CaCoFold structure	Rfam structure	covs	extra covs in CaCoFold	CaCoFold structure	ZWD structure	covs	other

		structure	cov_bps/bps	cov_bps/bps	obs/exp	structure	cov_bps/bps	cov_bps/bps	obs/exp	types
RT-16	RF03019	3	35/75	32/34	35/59.5	2	38/76	36/42	38/61.4	
DUF3800-I	RF02969	6	34/43	28/34	34/35.0	8	39/48	31/38	39/39.6	10
Betaproteobacteria-1	RF02932	2	31/49	29/36	31/28.0	2	31/49	29/36	31/28.6	
Transposase-1	RF03014	2	12/15	10/13	12/5.8	1	11/14	10/13	11/6.4	
nadA	RF03013	2	21/34	19/25	21/24.1					
DUF3800-V	RF02964	2	22/33	20/28	22/15.9	2	22/33	20/28	22/16.3	1
RT-5	RF03026	2	23/40	21/30	23/15.8	2	23/40	21/30	23/15.5	
ROOL	RF03087	80	199/222	119/172	199/202.6	25	200/218	175/182	200/200.2	10
MOCO RNA motif	RF01055	2	31/42	29/42	31/28.6					
radC	RF01754	2	24/42	22/35	24/29.8	1	26/56	25/40	26/26.2	
raiA	RF03072	8	44/61	36/60	44/41.8	1	46/59	45/63	46/41.5	
Pseudomonadales-1	RF03046	2	26/64	24/40	26/13.6	2	25/63	23/41	25/13.7	
SNORD10	RF01290	3	8/43	5/13	8/6.8					
RAGATH-5	RF02685	2	5/18	3/12	5/2.3	3	9/21	6/12	9/8.0	
MIR159	RF00638	1	6/66	5/26	6/34.5					
RAGATH-7-assoc	RF03105	1	6/29	5/34	6/1.5					
Thermales-rpoB	RF03109	2	4/28	2/16	4/0.3	3	5/37	2/16	5/0.5	7
cold-seep-1	RF03089	2	6/52	4/37	6/1.2	2	6/52	4/38	6/1.1	
Sr006	RF02889	1	4/34	3/30	4/0.0					
ceN104	RF01610	1	4/37	3/37	4/0.3					
snoR83	RF01227	1	3/34	2/33	3/0.7					
Collinsella-1	RF01700	2	5/53	3/52	5/1.8	2	5/53	3/52	5/0.2	
DUF1874	RF02953					1	24/33	23/30	24/15.0	
group-I-patch8						3	31/48	28/41	31/17.7	
MoCo						2	36/46	34/50	36/30.4	
pfl						1	17/26	16/29	17/15.2	
RAGATH-8	RF02687					2	26/63	24/63	26/14.3	
NiCo-AnGGAG	RF02683					2	11/32	9/31	11/2.8	

Type 10: Cross-covariations

		Rfam alignment				ZWD alignment				
RNA	Rfam ID	extra covs in CaCoFold structure	CaCoFold structure cov_bps/bps	Rfam structure cov_bps/bps	covs obs/exp	extra covs in CaCoFold structure	CaCoFold structure cov_bps/bps	ZWD structure cov_bps/bps	covs obs/exp	other types
c4-2	RF02944	5	29/33	24/24	29/26.2					
LOOT	RF03000	45	88/92	43/46	88/87.9	39	86/88	47/49	86/84.7	7
U5	RF00020	8	34/46	26/30	34/35.0					
tmRNA	RF00023	30	132/135	102/119	132/133.8					1,5
GA-cis	RF02987	5	22/29	17/21	22/24.4	4	22/28	18/22	22/24.0	
pan	RF01749	1	18/31	17/21	18/19.9	3	23/39	20/23	23/22.1	
sRNA-Xcc1	RF02221	2	18/25	16/20	18/12.5					7
Flavo-1	RF01705	30	45/57	15/19	45/50.5	40	59/78	19/21	59/66.2	
C4	RF01695	9	30/32	21/27	30/29.9					
wcaG	RF01761	4	21/35	17/22	21/23.3	4	20/35	16/23	20/17.3	
group-II-D1D4-2	RF01999	1	30/37	29/38	30/29.7	6	35/39	29/30	35/36.9	
RNase MRP	RF00030	1	53/87	52/74	53/48.9					
U11	RF00548	2	30/49	28/42	30/25.5					
DUF3800-II	RF02961	1	12/24	11/20	12/4.1	1	11/24	10/22	11/4.1	
U4atac	RF00618	3	17/39	14/30	17/14.3					
OTKONC	RF03053	8	21/54	13/30	21/3.5	8	21/54	13/34	21/3.7	
Clostridiales-3	RF02942	1	24/64	23/54	24/21.5	1	25/68	24/56	25/23.1	
snoMBII-202	RF00324	1	3/3	2/5	3/0.6					
isrK	RF01394	2	9/24	7/18	9/8.4					
CC2171	RF01867	9	15/74	6/29	15/9.3					7
twister-P1						4	30/31	26/26	30/30.2	
twister-P5						5	29/35	24/24	29/31.0	
pistol						6	30/36	24/25	30/31.7	2
Clostridiales-1	RF01699					1	65/72	64/69	65/53.3	
rRNA-patch1						17	78/83	61/66	78/76.8	
Rhodopirellula-1						1	17/19	16/18	17/14.3	
c4-patch1						5	33/35	28/32	33/33.4	
group-I-patch6						8	24/27	16/20	24/23.7	
Gamma-cis-1						4	21/31	17/28	21/10.1	
TD-1						4	19/47	15/36	19/5.2	

Type 11: Possibly alternative structures

		Rfam alignment				ZWD alignment				
RNA	Rfam ID	extra covs in CaCoFold structure	CaCoFold structure cov_bps/bps	Rfam structure cov_bps/bps	covs obs/exp	extra covs in CaCoFold structure	CaCoFold structure cov_bps/bps	ZWD structure cov_bps/bps	covs obs/exp	other types
GP20-b	RF03003	6	17/24	11/13	17/15.1	5	17/22	12/13	17/14.8	9
suhB	RF00519	10	21/37	11/13	21/21.7					
narK	RF03032	2	7/23	5/7	7/8.5	2	8/24	6/7	8/10.5	
DUF3800-IV	RF02963	1	12/39	11/36	12/1.7	1	12/39	11/36	12/1.8	
Termite-leu	RF01730	2	7/48	5/30	7/1.6	2	6/47	4/35	6/1.6	
Prevotella-2	RF03043	1	3/46	2/29	3/1.1					

Type 12: Additional covariations in SSU and LSU rRNA

		Rfam alignment				ZWD alignment				
RNA	Rfam ID	extra covs in CaCoFold structure	CaCoFold structure cov_bps/bps	Rfam structure cov_bps/bps	covs obs/exp	extra covs in CaCoFold structure	CaCoFold structure cov_bps/bps	ZWD structure cov_bps/bps	covs obs/exp	other types
LSU rRNA bacteria	RF02541	22	543/913	521/846	543/529.6					
LSU rRNA archaea	RF02540	12	466/914	454/786	466/540.6					
SSU rRNA bacteria	RF00177	1	240/473	239/462	240/238.4					
SSU rRNA eukarya	RF01960	4	208/432	204/447	208/249.4					
LSU rRNA eukarya	RF02543	8	385/894	377/872	385/494.0					
SSU rRNA microsporidia	RF02542	1	117/330	116/366	117/115.3					

Type 13: Multiple covariations not compatible with anything

		Rfam alignment				ZWD alignment				
RNA	Rfam ID	extra covs in CaCoFold structure	CaCoFold structure cov_bps/bps	Rfam structure cov_bps/bps	covs obs/exp	extra covs in CaCoFold structure	CaCoFold structure cov_bps/bps	ZWD structure cov_bps/bps	covs obs/exp	other types
STnc150	RF01402	22	22/98	0/22	22/3.1					
MIR824	RF00943	28	28/157	0/54	28/10.1					
P14	RF01669	4	4/37	0/36	4/0.1					

Type 14: Missalignment introducing spureous covariations

		Rfam alignment				ZWD alignment				
		extra covs	CaCoFold	Rfam		extra covs	CaCoFold	ZWD		

RNA	Rfam ID	in CaCoFold structure	structure cov_bps/bps	structure cov_bps/bps	covs obs/exp	in CaCoFold structure	structure cov_bps/bps	structure cov_bps/bps	covs obs/exp	other types
PhotoRC-II	RF01717	7	20/24	13/13	20/20.8	1	17/21	16/19	17/15.4	
pemK	RF02913	10	25/25	15/15	25/24.3	4	18/18	14/15	18/17.5	

Type 15: Low power; from zero or one covariation to one or two; inconclusive

		Rfam alignment				ZWD alignment				
RNA	Rfam ID	extra covs in CaCoFold structure	CaCoFold structure cov_bps/bps	Rfam structure cov_bps/bps	covs obs/exp	extra covs in CaCoFold structure	CaCoFold structure cov_bps/bps	ZWD structure cov_bps/bps	covs obs/exp	other types
HgcG	RF00064	1	3/79	2/57	3/0.2					
mir-190	RF00672	1	2/30	1/31	2/6.0					
snopsi28S-3327	RF00544	1	2/34	1/35	2/1.8					
mir-90	RF00785	1	2/8	1/36	2/0.1					
IRES HIF1	RF00449	1	2/79	1/37	2/5.2					
SNORA7	RF00409	1	2/38	1/37	2/2.6					
ceN101	RF01664	1	2/27	1/39	2/0.3					
SNORA3	RF00334	1	2/33	1/40	2/0.3					
STnc550	RF02081	1	2/77	1/53	2/1.4					
MNV 3UTR	RF02532	1	2/76	1/74	2/0.2					
Rev13	RF02737	1	2/93	1/112	2/0.0					
rli41	RF01473	1	2/95	1/128	2/0.2					
IsrR	RF01419	3	3/5	0/11	3/4.9					
K chan RES	RF00485	3	3/26	0/24	3/13.4					
GABA3	RF01803	1	1/17	0/21	1/1.7					
SpR19 sRNA	RF02447	1	1/18	0/30	1/2.5					
mir-393	RF02516	1	1/34	0/29	1/5.1					
snoZ188	RF00311	1	1/33	0/6	1/3.7					
rnk pseudo	RF01772	2	2/32	0/23	2/4.2					
fstAT	RF01797	1	1/14	0/18	1/1.4					
U54	RF00206	1	1/14	0/5	1/1.7					
snoR64	RF00267	1	1/21	0/11	1/0.6					
Spd-sr37	RF02419	1	1/19	0/12	1/1.8					
STnc130	RF02084	1	1/23	0/10	1/1.4					
SNORA42	RF00406	1	1/38	0/35	1/1.5					
Entero CRE	RF00048	1	1/20	0/12	1/3.5					
SSRC34	RF02526	1	1/36	0/36	1/2.4					
IRES Hsp70	RF00495	1	1/64	0/54	1/2.9					
mir-132	RF00662	1	1/34	0/16	1/5.5					
mir-143	RF00683	1	1/31	0/28	1/2.3					
SNORA69	RF00265	1	1/26	0/38	1/0.8					
SNORD125	RF01200	1	1/21	0/9	1/0.7					
porB	RF03042	1	1/3	0/7	1/0.1	1	1/3	0/7	1/0.1	
STnc630	RF02052	1	1/10	0/31	1/0.3					
TB6Cs1H1	RF01546	1	1/14	0/16	1/0.1					
PsrR1	RF02701	1	1/15	0/8	1/1.0					
RF site5	RF01093	1	1/11	0/16	1/0.2					
RsmY	RF00195	1	1/27	0/19	1/1.0					
snoR111	RF01228	1	1/21	0/31	1/0.3					
NRON	RF00636	1	1/24	0/64	1/0.8					
mir-338	RF00686	1	1/23	0/21	1/1.6					
snoTBR2	RF02786	1	1/16	0/33	1/0.2					
SNORD97	RF01291	1	1/31	0/13	1/0.7					
mir-365	RF00659	1	1/28	0/29	1/0.5					
Bp1 684	RF02421	1	1/23	0/16	1/0.4					
Rota CRE	RF00501	1	1/6	0/17	1/0.3					
snoR9	RF00065	1	1/36	0/18	1/0.3					
rpsL pseudo	RF01773	1	1/34	0/35	1/0.2					
IRES c-sis	RF00549	1	1/195	0/201	1/1.1					
mir-129	RF00486	1	1/36	0/25	1/0.5					
sX6	RF02225	2	2/87	0/68	2/2.0					
ceN100	RF01607	3	3/22	0/35	3/0.1					
mir-541	RF00777	1	1/25	0/30	1/0.7					
mir-246	RF00819	1	1/23	0/33	1/0.0					
SNORA67	RF00272	1	1/35	0/33	1/0.2					
scr4677	RF02673	1	1/66	0/36	1/2.3					
istR	RF01400	1	1/33	0/44	1/0.2					
snR4	RF01244	1	1/38	0/54	1/0.1					
MIR811	RF00882	1	1/84	0/81	1/0.5					
