# Supplementary material for: RNA structure prediction using positive and negative evolutionary information
Source: PLoS Comput Biol. 2020 Oct 30;16(10):e1008387. doi: 10.1371/journal.pcbi.1008387 (PMC7657543; doi:10.1371/journal.pcbi.1008387)
Supplement: Attachment — Submitted filename: response.pdf [file pcbi.1008387.s010.pdf]

August 24, 2020

Tamar Schlick and Nir Ben-Tal
Editors
PLOS Computational Biology

Dear Dr. Schlick and Dr. Ben-Tal,

Thank you for the reviews of my manuscript "RNA structure prediction using positive and negative evolutionary information" (PCOMPBIOL-D-20-00868)). I appreciate your response and the referees' expert comments. I have addressed all the referees' points making revisions as follows:

Reviewer #1

> *The paper is a real step forward, but it is hard to read and follow, not only because of the many cases discussed (too often maybe too cursorily). Some sentences state an observation but without a figure or enough precision to locate the observation in the structure. A thorough re-reading by the author would certainly help. I will give some examples below. Because of the importance of the work, I feel it is worth this additional effort to clarify some points. Some may sound trivial, but the recent controversy with long ncRNAs revealed the deep misunderstandings even among (computer) scientists working on RNA.*

I have revised all written observations about specific RNA structures to make their relationship to the corresponding figure more clear and direct. Figures 3, 4a, 5b (and supplemental Figures S5a,b,c and S6a) have been edited as well. See bellow for a detailed description of all cases. I also have revised the complete manuscript, and made additional clarifications at different places.

The reviewer also mentions three important references of pioneer studies that successfully used covariation to infer RNA structures unknown at the time. I have added those references to the Introduction.

> *At the onset, the author should state clearly what nucleotide exchanges are considered to indicate covariations. Only those pertaining to standard WC pairs and GU wobble? Some GA pairs do form pairs through their Watson-Crick edges. But AG can also form sheared pairs (trans Hoogsteen-Sugar edge) in which case AG is not structurally the same as GA, but such AG pairs do exchange with AA pairs interacting through the same edges. Or is any variation accepted? I do not think so from reading the text. This would be useful for understanding, for example lines 38-39.*

All variations are accepted. The reviewer's confusion points out to an important omission in the Introduction. I have added a paragraph before the above mentioned lines 38-39 indicating that the covariation measure used by R-scape (G-test) does not discard any nucleotide exchange observed in the alignment, and it is able to report other types of base pairs, should they present significant covariation.

In the Results section, under "The CaCoFold algorithm" which was the first reference to the G-test measure in the original manuscript, I have now made an additional clarification indicating that all exchanges count towards the covariation score.

Considering the reviewer's examples, A:G exchanges appearing in a WC basepair will be taken into account in the covariation score. However, a non Watson-Crick A:G pair that exchanges only to a A:A represents a change only in one of the two positions in the pair and would have no covariation. If the sheared pair would also allow other conformations involving a change in the A residue (from A:G), then all

exchanges would contribute to the covariation score. I don't know if that is the case, maybe the reviewer can tell.

Regarding lines 38-39, I have modified the sentence to make sure it is clear that both positive and negative pairs can be of any kind (WC or not) and present any type of variation.

> *I do not understand line 146.*

The operational definition of a helix has been edited for clarity.

> *Line 148. I understand the definition is operational but a one base pair helix is not meaningful since a helix is characterized by a twist angle between steps and the rise between two steps along the helical axis.*

The reviewer is right that in 3D we don't expect to find a helix consisting of just one basepair. However, it is possible to find a single basepair that "appears" to form a helix by itself. Consider the example of the SAM-I riboswitch in Figure 4b. There is a U:A basepairs (marked with '1') that covaries and appears to be a single basepair helix. That U:A basepairs actually stacks coaxially on P1 and in 3D it is just a continuation of P1, but from a 2D point of view it is a one basepair helix, and quite an important one!

One basepair helices also occur in higher layers of the algorithm constrained by just one non Watson-Crick covarying basepair. The algorithm will try to find a helix around the covarying pair, but if that is not possible, the layer will end with a "helix" with just one pair.

> *Lines 167-168. I find the use of "triplets" as confusing. Sometimes they are used to compare with triples in 3D and sometimes with alternative structures.*

In a CaCoFold structural annotation, I use "triplet" ("tr") to describe alternative helices that overlap with the main secondary structure. Alternative helices without overlap with the main secondary structure are labeled "pk". Thus, an alternative helix labeled "tr" includes at least one basepair with a base triplet, that is, one residues that is also involved in a different basepairs. I have updated the paragraph to clarify this definition.

Regarding the issue raised by the reviewer of triplets versus alternative structures, CaCoFold cannot distinguish whether an alternative helix labeled "tr" is a triplet or an alternative fold. The same happens with alternative helices annotated as "pk". They are likely to be pseudoknots, but as in the case of U2 snRNA with the non-overlapping alternative stems IIa and IIc, they could be also alternative folds. I have edited the paragraph to clarify this issue.

> *It is written that "nc" is used for 3D structures, but in Fig.5 for the tRNA CaCoFold, one finds "nc". That figure would need more explanations.*

The reviewer is correct, the annotation "nc" is only used for 3D structures. In Figure 5b, CaCoFold pairs that are confirmed by the crystal structure have been renamed using the 3D "nc" names so that the equivalences can be easily identifies. I see the confusion that that may have produced. I have modified Figure 5b by first removing the Rfam structure that was not adding much information, and by color coding the pairs in the CaCoFold and 3D structure. I have used blue for pairs common to CaCoFold and 3D, black for pairs unique to CaCoFold, and brown for pairs unique to the 3D annotation. The legend has also been

modified accordingly, as well as the corresponding text in the manuscript.

See also the legend of Fig.5 (where I do not understand the second sentence): "Four nc pairs and one pk pair with covariation support are found by CaCoFold and confirmed by the 1EHZ structure. Four base triplets (tr) and two pseudoknots (pk) have covariation support but have not been assigned to any basepair type by RNAView." For some nucleotides, there are several "tr" (e.g. R9) and one "nc".

The sentence has been re-written. I hope the changes described above concerning the definition of "tr" and the changes in Figure 5b also clarify the issue.

See also line 331, what does this mean? Only pairs with two H-bonds are reported by RNAView and many other similar programs (for example, as discussed below 3238 form a single H-bond contact, at most).

I feel fortunate that the reviewer is so knowledgeable about the tRNA structure, and all RNA structures in general. A note has been added regarding the nature of this pair both in the legend and in the main text. The sentence in line 331 and the whole paragraph regarding the tRNA structure has been edited with more details.

Fig. 5b. Among the possible positive pairs, those that fit the 3D structure are selected, but without knowing it, all those would be equivalent.

I think this statement shows a misunderstanding. All possible positive pairs that are not present in the Rfam structure are reported; not just those that fit the 3D structure. In the original Figure 5b, for clarity I had omitted four positive pairs between contiguous positions, which occur in the three loops. Those contiguous covariations are now present in the revised version. I have added a comment to that effect in the revised manuscript.

I hope the edited version of the tRNA paragraph and the legend of Figure 5b help avoiding the confusion regarding which positive pairs are depicted (all). In the edited Figure 5b, the positive pairs not in Rfam have been color coded: those that fit the 3D structure (in blue), those that don't (in black), those that are not due to RNA structure (orange), and those that appear in the 3D structure only (in brown).

I guess, this is the same for the pairs between the invariant G18G19 and invariant U55C56 where all possibilities are open and cannot be distinguished (pk1, pk2). I do not remember seeing a tRNA with U56 by the way.

Those positions are not completely invariant, otherwise they would not covary. For instance G18 (position 22 in the Rfam seed alignment) is 81.9% G, 9.9% A, 4.8% U and 3.4% C. In fact, the two adjacent positions, G18G19, covary with each other with E-value $1.75e^{-7}$. The same occurs for the other positions: the C56 (position 95 in the Rfam seed alignment) is 79% C 8% U 11% A, 2% G. In addition, C56 and R57 covary significantly with E-vale 0.0082.

The reviewer suggest, and I agree, that the pk_1, and pk_2 covariations may be secondary covariations relative to the pk and nc_7. Their E-values suggest that as well. I have added a mention to this in the manuscript.

What is very interesting (and this is why clarity and explanations are required) is that variations between regions are observed without those variations reflecting a pair or a contact but instead a concerted variation due to tRNA/protein interactions (like anticodon triplet and N73, see Lines 327) or global constraints on the highly functional anticodon loop (tr_4 between 36 and 38).

In Figure 5b, I have relabeled the former tr_4 as "2" and color code it in orange to indicate that this covariation is likely not due to RNA base pairing. The good thing is that the CaCoFold algorithm is able to incorporate these kind of positive pair that may carry information other than RNA basepairing at different layers without disrupting the RNA structure.

> *Nucleotides 32 and 38 form a single H-bond contact and the most frequent ones are C32_A38 or Y32_Y38 (and it is one or the other depending on the anticodon triplet).*

Noted, both in the figure legend and manuscript.

> *For the "triplets", nc_2 and nc_5, is it really meant as a triple or as an alternative fold? With a change in the definition of a helix this may be clarified.*

nc_2 and nc_5 are non Watson-Crick basepairs identified in the 3D structure (thus labeled in blue) and annotated as HWt and WSt respectively by RNAView. In the edited Figure 5b, 3D basepairs (that is, blue or brown pairs) report the name of the basepair family to which they belong.

> *All tRNAs were selected, but the type I and type II tRNAs do not have the same triples. Re-doing the calculations separately for each class might enhance the values (at least for type I).*

I appreciate the suggestion. I had not taken this variability into consideration. Indeed the Rfam alignment includes a subset of type II tRNAs, but does not annotate any basepairs in the variable loop (VL). By default, R-scape does not analyze any of the VL positions as they have more gaps than the default threshold of no more than 75% gaps. After increasing the gap threshold to include all columns in the analysis, the VL region shows three covarying base pairs, and because it covaries, CaCoFold adds the VL helix. Figure 5b has been modified to show the VL region, and this extended analysis.

As for the non Watson-Crick and tertiary interactions, the principal tertiary interactions common to both types (a-e, in Brennan & Sundaralingam) as well as two specific for type I (f and g) are present in the current analysis. I have noted the correspondences in the text. While it would be interesting to do as the reviewer suggest and separate the two types, I think it would be beyond the scope of this manuscript which already has analyzed many different structural RNAs.

> *Lines 172-173. I am not sure that the first part of that sentence is correct (the second yes).*

The sentence in question: "Lack of positive basepairs indicates lack of confidence that the conserved RNA is structural, and the proposed structure has no evolutionary support." has been replaced with: "Lack of positive basepairs results in a proposed structure without evolutionary support."

> *Overall, the comparisons with experimental data are not easy to follow. For the tmRNAs, there are more recent structures (one you cite somewhere but do not compare with Ramrath et al. Nature 2012; and a more recent one, Scient Rep (2018) 8:13587).*

Thanks for the additional reference. For the tmRNA Figure 3 in the manuscript, I looked for an image of the base pair annotation of the whole tmRNA molecule based on a 3D structure. Figure 4 from Kelley et al. provides that information in a concise and complete form. While different structures from different species or even mitochondrial instead of nuclear may bring small variations, here I am comparing to a consensus

structure, and I don't expect all details to be identical. If the reviewer considers that any of the structures in the other two publications add any structural element that I should consider, I will be happy to look into that. After comparing the three articles, I don't see that.

Line 217. Difficult to see that.

I added an asterisk in Figure 3 where the Kelley triplet is located, and the annotation is mentioned in the text.

Line 294. Difficult to see. You may want to look at the results from comparative analysis on RNaseP, A and B (JMB 279, 773 (98)).

Thanks for that reference. I wish I had drawn the RNaseP structure using the non-standard display which helps understanding the tertiary interactions. While I have kept the standard display, I have added labels to better identify the 5 regions of tertiary interaction in the Torres-Larios' figure. I have added boxes around the residues according to Massire (1998), and I have added the reference as well.

The extra covariations that CaCoFold incorporates are compatible with the reported tertiary interactions. I have removed the text that alluded to a mismatch. I had misinterpreted Torres-Larios' black line annotation are representing just two interacting residues, not two groups of residues as it is the case. I hope the boxes I have added, following Massire, help with that,

Lines 302, 303. What is the meaning of ???

Typo fixed. It means Figure S3.

Lines 314-315. State which pairs with numbers and show them clearly on the Figures.

I have split this sentence into four, one for each of the RNAs discussed (RNaseP B, group II, U5 and U3), with a more detailed description of the relevant covarying pairs and their role in shaping the structures. The key covarying pairs have been numbered in the corresponding Figures: S5(a), S5(b), S5(c) and S6(a), and referred to in the text.

Line 317. Y-Y covarying pair, which covariations are observed?

This is a C:U pair exchanging to a U:C pair in 46% of the 180 sequences in the Rfam seed alignment for U5 spliceosomal RNA (RF00020). The software RNAView using PDB structure 3jb9 (chain C) annotates this pair as "stacked". This information has been added to the manuscript.

Line 339. ??

Typo fixed. It means Figure S3.

Lines 381. What is precisely meant here? Is it in part due to the huge amount of tRNA sequences and the expected changes occurring in the helices? The "almost" is interesting: which ones are not involved in covariations?

There are few structural RNAs with so many covarying non Watson-Crick basepairs as tRNA. That follows in part from a combination of the following facts: there are many tRNA genes per genome, the tRNA genes

are highly variable in sequence, and despite the sequence variability, the structure is very conserved. tRNA alignments can have great quality while having sequence identity as low as 40%. In the tRNA Rfam seed alignment analyzed here, there are several positions that do not covary, for instance a conserved U in the anticodon hairpin loop, and an A at the 5' end of the D loop. The text has been edited with some more details.

On Fig. S7 I did not see Type 12.

Type 12 corresponds to the small and large subunits of rRNA. It would have been difficult to draw these large structures, and as the reviewer mentions earlier, there are many (probably too many) structures already described in this work. I expect that the specific changes introduced by the additional positive pairs in SSU and LSU rRNA will fall into several of the 1-11 Types. That is why I put them in a class or their own. I hope the Rfam curators will soon look into those differences, but I decided not to present any results about rRNA in this manuscript. I believe that with the other more than 20 structures presented, I have shown the case for CaCoFold.

Reviewer #2

1. It is probably worth pointing out that the majority (97%98%) of the rRNA secondary structure had been inferred before the crystal structure was published thanks to covariation analysis [1].

Point noted in the Introduction, and reference [1] added.

2. It was unclear to me what was meant by "negative information". The MS defines this as basepairs that "show variation but not significant covariation". So, does this include column pairs that include "U:A & U:G" type of variation, that admittedly is not strictly covarying, but may be structurally consistent in the right context. Some careful definitions of positive and negative information would help the reader immensely.

Thank you for this observation, which was also raised by reviewer #1. A new paragraph (starting at new line 35) has been added to the manuscript to clarify this point. In a nutshell, structural consistency is not evidence of a conserved RNA structure (many structurally consistent helices can be form for any RNA, even random ones), and the covariation measure used by R-scape does reward structural consistency. Positive (ie, significantly covarying) basepairs don't have to be consistent with standard Watson-Crick basepairs, and negative pairs (ie pairs that show variation but not covariation) could include instances of consistent base pairs.

3. The use of a -log(E-value) scores concerns me slightly. With BLAST E-values this doesn't have a very good history (e.g. https://genomebiology.biomedcentral.com/articles/10.1186/s13059-015-0721-2), as directly using bit-scores can be a more accurate measure. I wonder if the author considered experimenting with directly using the G-score for the first layer?

I have, and there is no difference. Notice that this score is only used by the maxCov algorithm just to decide on the order in which subsets of nested covarying pairs get incorporated in the folding cascade, and all covarying basepairs will end up in the structure eventually.

For the R-scape one-set test (default), the covariation score and its E-value are directly proportional to each other, and there is no difference at all between using one or the other. For the R-scape two-set test (option -s), the only difference may be for instance on which of the two stems forming a pseudoknot is included as

part of the main secondary structure and which is added at a subsequent layer. That does not change the final structure, just the visual arrangement of it.

4. I'm curious about the types of Rfam families that could be improved by CaCoFold. Were these predominantly riboswitches and ribozymes, or other types? It's disappointing the the 5S rRNA has not been updated in Rfam. A number of papers have now pointed out flaws in this e.g. Rivas, Clements & Eddy (2017) and Gardner & Eldai (2015). Have the curators of Rfam been alerted to this issue?

The whole list of Rfam families for which the CaCoFold structure includes more covarying basepairs is given as a table in the supplemental materials, and it is not just riboswitches and ribozymes. It includes a total of 314 different RNA families (319 alignments). The supplemental table groups all families by the Type of modification that the CaCoFold structure introduces relative to the given consensus structure. Table 1 in the manuscript list the subset of 21/319 families that have 3D structures. In addition to riboswitches and RNaseP, it includes: SRP RNA, tmRNA, group-II intron, U2, U3, U4, tRNA, 5S rRNA, and 6S RNA.

The reviewer mentions the 5S rRNA structure. I believe the Rfam curators are aware, and I anticipate that the next Rfam version will include the correct 5S rRNA secondary structure.

5. Do the improved consensus structures improve the performance of the corresponding covariance models for homology search? Or are these robust to errors in alignments and structures?

That is an interesting point. I believe it will be the case that an improved consensus structure will results in better homology recognition, but I have not tested that for this manuscript. This could be a good subject for a subsequent manuscript. An infernal model using an improved structure should result in better homology detection. The extent of the gain will depend on the RNA. It is also true that methods like infernal are robust against errors both in the structure or the alignment. In some cases, the same homologs will be detected but with better scores, and in some cases the increase in score could be the difference between being detected or not.

6. Regarding the use of PDB structures. Presumably the consensus structure is generally a subset of basepairs parsed from 3D structures. I did see this explicitly mentioned in the MS.

It is not necessarily the case that the Rfam consensus secondary structure for an RNA with crystal structures is derived from the 3D structures. In fact, in the majority of cases it is not. I cannot find which part of the manuscript may have lead to such assumption.

The Rfam team is working hard at this, and I hope that soon the structures of RNAs with 3D information will be derived directly from those, but that is not the case up to Rfam v 14.2.

A "Y-Y" covariation is mention on pg 15. Presumambly this is a non-canonical interaction?

It is a C:U pair exchanging with U:C. See response to reviewer #1 for more details, added to the manuscript as well.

The "??" throughout the manuscript suggest missing references?

Typo fixed. It means Figure S3.

> *Figure 3: "Transfer-messenger RNA" spelled incorrectly*

Typo fixed. Thanks for catching that.

> *Installing the supplement: ...v1.5.6 seems to install fine though.*

With some warnings, I believe the code installs fine in standard platforms, while it only requires a total of three commands for the whole process to work (./configure; make; make install;). Some of the code used by R-scape is not my own (R2R, RNAView, FastTree, hmmer...), and I don't completely control their behavior.

Reviewer #3

> *I do not believe, however, that this result allows to conclude that conserved structures derived from sequence alignments without CaCoFold is unreliable.*
> *In the Summary, the author contends that predicting an overall consensus structure consistent with a set of individual inferred pairs and non-pairs remains a problem. How can this be since secondary structure prediction methods are reliably and systematically used to find structures under accumulated experimental constraints, and in particular chemical probing constraints that exactly predict pairs and non-pairs (e.g. SHAPE)?*

Despite many publication doing just that, a secondary structure prediction combined with experimental chemical probing is not evidence of a conserved RNA structure. Any sequence including random sequences will have a chemical probing signature that combined with a secondary structure prediction program will produce a plausible structure. Using just chemical probing and a prediction method, one cannot address the question of whether a conserved structural RNA exists or not. Besides, even if actually there is a conserved RNA structure, the one obtained by this method will likely be at least incomplete as this method usually do not identify pseudoknots or base triplets.

It is important to notice that SHAPE and other chemical probing methods do not "predict pairs and non-pairs", they give a score per residue but not pair information, and the predictions made with such data are far from "exact".

> *In Results, why X-ray crystallographic structures are such a great reference? After all, these structures are derived from crystals often obtained in denatured conditions.*

X-ray crystallography, as well as NMR and cryo-EM are to this day the most reliable methods and considered the state of the art for determining the structure of an RNA or a protein.

Crystals are not obtained (as the reviewer suggests) in denatured conditions which result in no structure. On the contrary, crystallographers carefully reproduce the native conditions (RNA concentration, temperature, salt and precipitants) that result on RNA molecules that are folded and conformationally homogeneous. Many different biological assay are used to assure that is the case, as well as to prevent other artifacts that could result on missfolded molecules. (See "Strategies in RNA Crystallography" Methods in Enzymology Volume 469, 2009, Pages 119-139.)

In this manuscript, I also argue with the example of the U2 spliceosomal RNA (Figure 5c) that covariation analysis on aligned homologs is a good complement to crystallography when alternative structures are present, since crystallography can only report one of the possible structures, but not the existence of both,

while covariation my indicate that that is the case.

tmRNA example: In Rfam, the R-scape structure has "103 out of 127 basepairs are significant at E-value=0.05". In the manuscript, we read "121 significantly covarying basepairs"; 69 identified in layer 1. Where are the discrepancies from?

The second quote is accurate. The R-scape analysis (not using the Rfam annotated structure) reports 121 covarying basepairs. Also reported in Figure 3d, the CaCoFold structure has 139 basepairs and includes all 121 positive basepairs. In order to include all 121 positive pairs, CaCoFold requires 6 layers. The number of positive pairs accounted for in each layer is given in Figure 3c. The sum of the pairs in all 6 layers is 121.

I believe the first quoted sentence in not from this manuscript, but from the Rfam website (`https://rfam.xfam.org/family/RF00023#tabview=tab3`),

"R-scape optimised structure: 103 out of 127 basepairs are significant at E-value=0.05"

Instead, in Figure 3, I report 121 positive pairs out of 139 basepairs.

Those two results are different for two reasons:

- **A different version of CaCoFold.** Rfam 14.1 used an early prototype of the CaCoFold algorithm, different from the current version in several respects. One of the most important differences is that in the Rfam prototype, once a covarying basepair had been added to the structure, the two involved residues were forbidden from basepairing at all at any subsequent layer. In the current version, they are only forbidden to pair to each other, but could pair to other residues. This behavior is described in a paragraph in section "The cascade folding algorithm" starting at line 155 in the revised version of the manuscript.
- **A different mode of R-scape.** R-scape has two main modes to produce a list of significantly covarying pairs. Mode (1) tests a proposed structure; Mode (2) ignores any given structure, and tests all possible pairs as equivalent. These two modes are described in detail in the Methods section.

  Depending on which R-scape mode is used, the CaCoFold prediction may be slightly different. Rfam uses Mode (1) as it is trying to use CaCoFold to improve the existing consensus structure. On the other hand, in this manuscript and in order to show the behavior of CaCoFold alone, the tmRNA analysis uses Mode (2) where only the alignment is taken into account.
  This is mentioned inn the manuscript in the section about the tmRNA analysis, in the Methods, and in Figure 3 (in orange).

It would help to compare the new result with that from R-scape to appreciate the plus values of CaCoFold versus a conserved structure derived from sequence alignement alone. For instance, are the 103 basepairs from R-scape (from Rfam) are true positives in the Kelley and colleagues EM structure?

The tmRNA structure reported in Figure 3 is derived from the sequence alignment alone. As I just mentioned above, that fact is stated in the text and figure. I have re-written slightly the text to make that fact even more clear.

The 103 positives reported in the Rfam page are a subset of the 121 reported in Figure 3. The 121 positive pairs and the rest of the 139 basepairs in the tmRNA CaCoFold structure are discussed in relation to the

Kelley structure in detail in Figure 3.

The structure is compared to the one obtained from RNAalifold. Could it be compared to the structure obtained with RNAstructure, common to three or more tmRNA sequences?

The point of comparing CaCoFold to other standard methods (Figure S1) is two fold. First, to show that CaCoFold in addition to providing a structure, it adds the important additional information of which basepairs or helices can be trusted because they have RNA covariation signal and which can only be considered predictions because they lack covariation support. That is the difference between Figure S1(a) and S1(b). The second point is that while most methods cannot deal with pseudoknots or base triplets, CaCoFold does, and it does very well at it, and can report complex pseudoknotted structures such as that of tmRNA. That is the difference between Figure S1(b) and Figure 3f. I could have used either RNAstructure or RNAalifold to show those two points, but it did not seem necessary to show both.

In the Introduction, I dont understand what the author means by Structure and biological function can be closely related . Why can? Is there any case where this is not the case?

I replaced “can be” with “usually are”.

In some cases, knowing the structure of an RNA almost immediately leads to making a good guess about its function, as it was the case for riboswitches. For other RNAs though that is not the case. For instance, we still have no idea about the function of the Vault RNA even if its structure is pretty well determined, and it has been known for some time (van Zon et al., The vault complex. Cell Mol Life Sci, 2003).

In (3) Filtering of alternative helices, the arbitrary values of 15 basepairs and 50% overlap with other bases with another helix should be justified.

I have added a sentence in the text to clarify this point. The reason is completely empirical; otherwise, the number of found alternative helices without any covariation support is very large. It is important to notice that this restriction applies only to alternative helices that have no covariation support. Alternative helices with at least one covarying basepair are always reported.

I hope this revised manuscript addresses the reviewers’ points, and I thank you again for the helpful comments.

Sincerely,

Elena Rivas, Ph.D.
