# Supplementary material for: RNA structure prediction using positive and negative evolutionary information
Source: PLoS Comput Biol. 2020 Oct 30;16(10):e1008387. doi: 10.1371/journal.pcbi.1008387 (PMC7657543; doi:10.1371/journal.pcbi.1008387)
Supplement: Attachment — Submitted filename: response.pdf [file pcbi.1008387.s012.pdf]

September 16, 2020

Tamar Schlick and Nir Ben-Tal
Editors
PLOS Computational Biology

Dear Dr. Schlick and Dr. Ben-Tal,

Thank you for the reviews of my manuscript “RNA structure prediction using positive and negative evolutionary information” (PCOMPBIOL-D-20-00868)). Please find attached a new version in which I address the additional comments of referees #1 and #3 as follows:

Reviewer #1

> *For me, a single base pair helix remains an oxymoron. Mathematically, a helix is a rotation angle between two objects and a translation along an axis. Such single base pairs are tertiary base pairs that happen to form WC and are in the usual helical conformation (they usually start or close a helix). Because they appear to conform to pairs forming a helix in stacking continuity, they could also be considered as belonging to the defining helix. The example given by the author (SAM I riboswitch) shows clearly that the additional A-U pair extends helix P1 and could be considered to belong to P1 (covalent continuity in chains is not a prerequisite for a 3D helical conformation). BTW the figure 4b shows it stacking with P4 unlike the Xtal drawing next to it.*

I understand the reviewer’s apprehension. I should have used a word other than “helix” which has the specific meaning given by the reviewer of involving a rotation and a translation between two base pairs. The concept I wanted to convey is “a block of contiguous or quasi-contiguous base pairs in nested conformation”. This concept is used in the filtering step of the algorithm, for which it does not matter whether the number of base pairs in the “block” is larger of one or not. In the text, I have tried to state this overuse of the term “helix”.

Regarding the reviewer’s observation about Figure 4b, the CaCoFold display is not smart enough to know how to stack that A:U base pair. It draws it automatically with the only consideration of page clarity (using the software R2R). However, once you see that the A:U pair is contiguous to the last C:G in P1, it is easy to guess that it stacks coaxially with P1, and the fact that both base pairs involved covary adds support to the hypothesis.

> *G18G19 are invariant in bacterial and cytoplasmic tRNAs. If the alignment includes mitochondrial tRNAs where the D- and T-loops are either absent or widely different with their structures still not known (and not structurally homologous). This would be the same for C56. Slight misalignments (due for example to insertion/deletion of one nt for example) would also lead to apparent variations. In tRNA pseudogenes, one can also observe such variants and they should have been pruned out of the alignment. I think the Rfam tRNA seed contains results from tRNAscan and also mitochondrial sequences (the evolution and structure of which are different from cytoplasmic ones). I would remove from the Rfam database all sequences where the GG is not there and re-run. They are either pseudogenes or from mitochondria and in either case covariations or correlations found using them are meaningless and may detract from real ones.*

Thanks for the remark about tRNA pseudogenes. Pseudogenes can be a problem in any covariation analysis as they can weaken the covariation signal, or produce spurious covariations. I have noted the possible issue with tRNA pseudogenes in the manuscript.

Misalignments is another source of confounding covariation. In this manuscript, I have observed and noted

the effect of two misaligned contiguous positions in the stop codon of the tmRNA.

Line 368: the sentence is not correct; all tRNAs have a V loop but only class II have a V region that forms an additional helix.

Thanks for the correction. The sentence has been modified accordingly.

Group II introns in Rfam form only a small fragment.

Thanks for the observation. In the text, I have clarified that the Rfam family RF02001 used in the group-II intron analysis correspond to a fragment named D1D4-3.

Reviewer #3

The author did not understand my first comment. SHAPE produces flexibility signals which are translated in secondary structure prediction programs by propensities of nucleotides to participate or not in base pairs; therefore equivalent to pairs and non-pairs. These programs always find acceptable solutions, and therefore they do not represent the bottleneck of RNA structure prediction. Besides, some of these programs also predict tertiary interactions (not referred to by the author). However, I agree with the author that a structure thus obtained rather corresponds to the structure under experimental observation than a structure supported by covariations.

Thanks for the clarification about the SHAPE comment. To address this comment, I have modified a paragraph in the introduction with a mention to the use of positive and negative propensity information when chemical probing data is incorporated into methods to infer an RNA structure.

In fact, these two form of inference are not mutually exclusive. One can devise methods to predict RNA structure using both covariation and chemical probing.

In my initial comment on X-ray crystal structures, I said denatured, but what I meant is more not under physiological conditions. I am sorry for this lack of precision. Therefore, I do not agree with the author that "crystallographers carefully reproduce native conditions". I would rather say that the crystallographers, although they try to reproduce the native conditions, aim at obtaining crystals, whether the physiological conditions are respected or not and they seldom are. The case of U2 spliceosomal RNA is interesting because the aligned homologs lead to interactions that differ from the crystal. Q1. Does the covariation data substantiate both alternatives, or one of them only?

Thanks for the clarification about the crystallography comment.

Q1: Yes, the covariation signal in U2's Rfam seed alignment supports the two alternative helices (see middle structure in Figure 5c). Stem IIa and Stem IIc are the two alternative helices. Stem IIa has six covarying base pairs, and Stem IIc has two.

Now, about using RNAStructure rather than RNAalifold: I understand that it is not necessary to show both if indeed it leads to the same conclusions. Q2. Is that the case ?

Q2: It turns out, RNAstructure does not have any tools to produce a consensus structure from an RNA alignment. I was surprised by that; I had assumed it did. Therefore, RNAalifold is the best tool in the field for that task, and the obvious method to compare to CaCoFold. That opinion was shared with me by Dave Mathews (the creator of RNAstructure himself) when I asked him about which RNAstructure tool I should use. Any comparison of CaCoFold (or RNAalifold) to RNAstructure would not be fair as RNAstructure does not use covariation information directly.

I hope this revised manuscript addresses the reviewers' last comments.

Sincerely,

Elena Rivas, Ph.D.
