# Supplementary material for: RNA structure prediction using positive and negative evolutionary information
Source: PLoS Comput Biol. 2020 Oct 30;16(10):e1008387. doi: 10.1371/journal.pcbi.1008387 (PMC7657543; doi:10.1371/journal.pcbi.1008387)
Supplement: Attachment — Submitted filename: PLOS_Comp_Rivas.docx [file pcbi.1008387.s011.docx]

The author very seriously and systematically addressed the points raised by the referees.

A couple of points that the author might want to consider or not.

- For me, a single base pair helix remains an oxymoron. Mathematically, a helix is a rotation angle between two objects and a translation along an axis. Such single base pairs are tertiary base pairs that happen to form WC and are in the usual helical conformation (they usually start or close a helix). Because they appear to conform to pairs forming a helix in stacking continuity, they could also be considered as belonging to the defining helix. The example given by the author (SAM I riboswitch) shows clearly that the additional A-U pair extends helix P1 and could be considered to belong to P1 (covalent continuity in chains is not a prerequisite for a 3D helical conformation). BTW the figure 4b shows it stacking with P4 unlike the Xtal drawing next to it.
- G18G19 are invariant in bacterial and cytoplasmic tRNAs. If the alignment includes mitochondrial tRNAs where the D- and T-loops are either absent or widely different with their structures still not known (and not structurally homologous). This would be the same for C56. Slight misalignments (due for example to insertion/deletion of one nt for example) would also lead to apparent variations. In tRNA pseudogenes, one can also observe such variants and they should have been pruned out of the alignment. I think the Rfam tRNA seed contains results from tRNAscan and also mitochondrial sequences (the evolution and structure of which are different from cytoplasmic ones). I would remove from the Rfam database all sequences where the GG is not there and re-run. They are either pseudogenes or from mitochondria and in either case covariations or correlations found using them are meaningless and may detract from real ones.
- Line 368: the sentence is not correct; all tRNAs have a V loop but only class II have a V region that forms an additional helix.
- Group II introns in Rfam form only a small fragment.
